# ¿Perdonar? Imposibilidad de tramitar daños e injusticias en mujeres víctimas de violencia sexual*

**DOI:** 10.15649/cuidarte.2536

**Published:** 2023-09-02

**Authors:** Mara Mercedes Osorno-Navarro, Isabel Cristina Posada-Zapata

**Affiliations:** 1 Doctoranda en Salud Pública Universidad CES, Magister en Enfermería, Especialista Docencia Universitaria, Enfermera, Profesor Asociado Universidad de Sucre. Colombia Email: osorno.mara@uces.edu.co Universidad de Sucre Universidad de Sucre Colombia osorno.mara@uces.edu.co; 2 Doctora Ciencias Sociales, Niñez y Juventud Fundación Centro Internacional de Educación Y Desarrollo Humano-CINDE/Universidad de Manizales, Magister en Salud Pública Psicóloga, Profesora Universidad de Antioquia Colombia. E-mail: isabel.posada@udea.edu.co Universidad de Antioquia Universidad de Antioquia Colombia isabel.posada@udea.edu.co

**Keywords:** Violencia Sexual, Significado, Mujer, Víctima, Conflicto Armado, Colombia, Sexual Violence, Meaning, Women, Victim, Armed Conflict, Colombia, Violencia Sexual, Significado, Vítima, Mulher, Conflito Armado, Colombia

## Abstract

**Introducción:**

La violencia es un problema de salud pública que cada día se ha incrementado, por ello, es importante fortalecer las capacidades de vigilancia y gestión del riesgo, y de acabar con las agresiones, respetando, protegiendo y garantizando los derechos de mujeres y niñas para que puedan alcanzar todo su potencial; haciendo un particular hincapié en la indagación de dispositivos que permitan poner fin a todas las formas de discriminación y violencia en contra de ellas.

**Objetivo:**

Reconocer los significados sobre el perdón construidos por las mujeres víctimas expuestas al conflicto armado en Montes de María, San Onofre Sucre como consecuencia de las violencias sexuales ejercidas por paramilitares.

**Materiales y Métodos:**

Se utilizó un enfoque cualitativo a través de la Teoría Fundamentada y el Interaccionismo Simbólico para generar la teoría, a partir de los significados que emergen de la interacción entre las personas y su entorno.

**Resultado:**

La teoría sustantiva resultante evidenció las características de la experiencia de la violencia sexual en la mujer; la cual simboliza desazón, exilio, destrucción de sus metas y proyectos, además de la dificultad para perdonar debido a la magnitud del daño causado, y al incumplimiento de la legislación que debía proteger a la mujer víctima de tal violencia.

**Conclusión:**

Dentro de los significados de las violencias sexuales se encontró que las mujeres tienen dificultad para perdonar, por la imposibilidad de olvidar el acto de horror cometido contra ellas; aducen que, para entrar en el camino del perdón y la reconciliación, los vándalos deben revelar la verdad sobre los actos cometidos, así como mostrar un arrepentimiento genuino.

## Abstract (en)

**Introduction:**

Violence is a public health problem that is growing every day; therefore, it is important to strengthen monitoring and risk management capabilities and to put an end to aggressions by respecting, protecting, and guaranteeing the rights of women and girls so that they can reach their full potential. Particular emphasis is placed on seeking mechanisms to end all forms of discrimination and violence against women and girls.

**Objective:**

To recognize the meanings of forgiveness constructed by women victims of the armed conflict in Montes de María, San Onofre Sucre, as a consequence of sexual violence perpetrated by paramilitaries.

**Materials and Methods:**

A qualitative approach of grounded theory and symbolic interactionism was used to develop a theory based on the meanings that emerge from interactions between people and their environment.

**Results:**

The resulting substantive theory demonstrated the characteristics of women's experience of sexual violence, which symbolizes distress, exile, the destruction of their goals and projects, as well as the difficulty of forgiveness due to the magnitude of the harm caused, and the non-compliance with legislation that should protect women victims of such violence.

**Conclusion:**

Among the meanings of sexual violence, it was found that forgiveness is difficult for these women because it is impossible to forget the act of horror committed against them. They argue that, in order to enter the path of forgiveness and reconciliation, offenders must reveal the truth about the acts committed and show genuine repentance.

## Resumo

**Introdujo:**

A violencia é um problema de saúde pública que aumenta a cada dia, por isso é importante fortalecer as capacidades de vigilancia e gestao de riscos, e acabar com as agressóes, respeitando, protegendo e garantindo os direitos das mulheres e meninas para que possam atingir todo o seu potencial; com particular enfase na investigado de dispositivos que permitem acabar com todas as formas de discriminado e violencia contra elas.

**Objetivo:**

Reconhecer os significados do perdao construidos por mulheres vítimas do conflito armado em Montes de Maria, San Onofre Sucre, como consequencia da violencia sexual perpetrada por paramilitares.

**Materiais e Métodos:**

Foi utilizada uma abordagem qualitativa por meio da Grounded Theory e do Interacionismo Simbólico para gerar a teoria, com base nos significados que emergem da interado entre as pessoas e seu ambiente.

**Resultado:**

A teoria substantiva resultante evidenciou as características da vivencia da violencia sexual em mulheres; que simboliza o desespero, o exílio, a destruido de seus objetivos e projetos, além da dificuldade de perdoar pela magnitude dos danos causados, e o descumprimento da legislado que deveria proteger as mulheres vítimas dessa violencia.

**Conclusao:**

Dentro dos significados da violencia sexual, constatou-se que as mulheres tem dificuldade em perdoar, devido a impossibilidade de esquecer o ato de horror cometido contra elas; eles argumentam que, para entrar no caminho do perdao e da reconciliado, os vándalos devem revelar a verdade sobre os atos cometidos, bem como mostrar arrependimento genuíno.

## Introducción

Los informes de la OMS en nombre del Grupo de Trabajo interinstitucional de las Naciones Unidas registran sobre la violencia contra la mujer, en todo el mundo, que una de cada tres mujeres (un 30%) ha sufrido violencia física y/o sexual por su pareja o violencia sexual por alguien que no era su pareja o ambas. A nivel mundial, hasta el 38% de los asesinatos de mujeres son cometidos por sus parejas. Además de la violencia de pareja, el 6% de las mujeres de todo el mundo refieren haber sufrido agresiones sexuales por personas distintas de su pareja, aunque los datos al respecto son más limitados. La violencia de pareja y la violencia sexual son perpetradas en su mayoría por hombres contra mujeres^1^.

Los impactos de la violencia en la salud de las personas han variado según las dinámicas temporales y espaciales del conflicto armado interno. En los periodos en los que la intensidad y la degradación del conflicto armado fueron mayores, hechos como la violencia sexual, la tortura, las masacres, los atentados contra la vida, los ataques a bienes protegidos^2^, los atentados indiscriminados y las minas antipersona causaron afectaciones sobre la salud mental y daños en el cuerpo que, en el corto y largo plazo, limitaron la autonomía de las personas, les impidieron realizar sus actividades y cambiaron su manera de relacionarse con el mundo. Las mujeres han estado expuestas de forma permanente a las condiciones de la guerra marcadas por el miedo, la incertidumbre, la ansiedad y las pérdidas crecientes de seres queridos que afectaron la salud de miles de ellas^3^.

Todos los grupos armados han utilizado la violencia sexual como una estrategia de guerra, particularmente en contra de niñas, adolescentes y mujeres. Según datos del Observatorio de Memoria y Conflicto del CNMH, desde 1961 y hasta 2021, se han documentado 15.236 víctimas de violencia sexual. De estas, el 90,5 % de las víctimas fueron mujeres, en especial contra las mujeres afrodescendientes e indígenas^4^. El RUV, por su parte, reporta, a enero de 2022, 32.407 personas víctimas de delitos contra la libertad y la integridad sexual^5^.

Las mujeres de este país, puntualmente las de la zona rural y con precarios recursos socioeconómicos, con deficientes niveles de educación, son sujetas a diferentes maneras de violencia: el desplazamiento forzado interno, el homicidio, el reclutamiento forzado, la desaparición y tortura, que integran prácticas generalizadas de los actores armados, para ejercer control sobre los territorios y característicamente “sobre las mujeres^6^”.

En correspondencia, Saúl Franco, resalta que la violencia sexual ha sido un arma de guerra destructora, callada y reiterativa. Con diferentes características e intensidades, todos los involucrados del conflicto armado colombiano la han utilizado, principalmente en contra las mujeres. Si este fenómeno no se destituye, no se atiende y no se hace justicia a sus víctimas, los pactos serán incompletos y la paz dudosa^7^.

Y es así como el conflicto armado se tomó a los Montes de María (MM), región ubicada en la parte central de los departamentos de Sucre y Bolívar en el caribe colombiano, sobre una prolongación de la serranía de San Jerónimo, uno de los tres ramales que divide la Cordillera Occidental. Las tierras de los MM son las más fértiles del país. Esta región se divide en cinco zonas, la zona del golfo de Morrosquillo -la correspondiente a este estudio-, con importancia geopolítica, para la intercomunicación entre diferentes zonas del norte del país. Comprende el municipio de San Onofre (SO), Tolú, Tolú Viejo -región relacionada con presencia de narcotráfico-, organizaciones criminales usadas para la prestación de servicios de seguridad, y actos delincuenciales, por inversionistas ilegales en 1990. El conflicto armado, las masacres, el uso de la fuerza contra la población civil originaron procesos de desplazamiento forzado principalmente en San Onofre en 1999 y 2007 con una alta magnitud, que agravaron los índices de pobreza, y delincuencia, con apropiación y control del territorio^8^.

En estos territorios, la violencia sexual es un tema poco investigado en el marco del conflicto armado. Las violencias sexuales se presentan como una situación imperante y relevante de la Salud Pública, ya que, desde la búsqueda de sus causas, pero sobre todo los significados, de las maneras de afrontarlas, se puede obtener información sustentada para optimizar la garantía, desarrollo y cumplimiento de los derechos de las mujeres y formular planes particulares que incluyan las variables de su situación geográfica y sociocultural con un enfoque diferencial.

Teniendo en cuenta lo anterior, esta investigación tuvo como objetivo reconocer los significados construidos sobre el perdón por las mujeres víctimas expuestas al conflicto armado en Montes de María, San Onofre (Sucre) como consecuencia de las violencias sexuales ejercidas por paramilitares (1990-2005).

## Materiales y Métodos

En esta investigación fue indispensable utilizar el enfoque cualitativo con la teoría fundamentada, con este método, la recolección de datos, el análisis y la teoría que surgirá de ellos, guardarán estrecha relación entre sí^9^”. El fin determinado de este método estableció el proceso de comparación constante entre los diversos testimonios de las mujeres participantes, y permitió, a partir de la codificación abierta, la construcción de categorías conceptuales y patrones de acción descriptivos que generaron el desarrollo de teoría.

Las interlocutoras eran escogidas en dos organizaciones de mujeres agredidas sexualmente del municipio, tales como: Red de Mujeres Constructoras de Paz y ASOMUSAN (Asociación de Mujeres de San Onofre). En total, las dos asociaciones cuentan con alrededor de 176 integrantes, de las cuales el 80% son víctimas de violencia sexual. Las mujeres seleccionadas residían en San Onofre durante la investigación, o en sus corregimientos tales como: Libertad, Rincón del Mar, Berrugas, Berlin, Plan Parejo, Buenos Aires y La Barcés. Las participantes fueron mujeres mayores de edad, que afirman haber sido abusadas sexualmente por paramilitares (periodo 1990 a 2005). Dichas mujeres fueron seleccionadas haciendo uso de un muestreo teórico, procedimiento más conveniente para este tipo de investigaciones. Era la propia investigación la que le decía a la investigadora en qué momento la información comenzó a ser repetitiva, o el fenómeno que se pretendió comprender estaba claro^10^.

En síntesis, el muestreo teórico permitió ir eligiendo personas que reunieran las características necesarias para aumentar el nivel de comprensión de las categorías que fueran emergiendo durante el análisis. A medida que surgió la necesidad de obtener más información se escogieron nuevas participantes, las cuales respondieron a las necesidades del muestreo teórico, llegando a una muestra total de 11 interlocutoras, momento en el que se obtuvo la saturación teórica, es decir, un estado en el que las categorías ya no encuentran elementos nuevos, ni en sus propiedades ni en sus dimensiones.

La investigación fue aprobada por el Acta N125 del Comité de Ética en Investigación en seres humanos de la Universidad CES de Medellín.

En la primera fase, la recolección inició en el año 2019 con la realización de las 4 primeras entrevistas semiestructuradas, a profundidad. Las narraciones de las mujeres fueron grabadas y posteriormente transcritas por una persona externa, los textos fueron luego revisados para garantizar su fidelidad respecto a los audios. En ese año se presentó una situación de orden público, los actos de violencia y los asesinatos perpetrados por grupos criminales amedrentaron a todo un pueblo con miedo de revivir épocas pasadas, motivo por el cual la investigadora, siguiendo los consejos de la población, decidió no continuar con el proceso de recolección de información en el municipio.

En el año 2020, por los continuos episodios de violencia ocurridos en San Onofre, se determinó establecer el traslado de las participantes hacia el municipio de Sincelejo cada vez que se requiriera una entrevista, con el fin de proteger la integridad física de todos los involucrados en la investigación (febrero 2020).

En el análisis la investigadora interpretó la narración teniendo en cuenta el contexto de las interlocutoras, sus comportamientos, actitudes, gestos, lágrimas y silencios para lograr una mayor comprensión de las situaciones narradas^11^. Por otra parte, las transcripciones de las entrevistas se realizaron en Word, estas fueron divididas por frases con el fin de formar conceptos; asimismo, se incluyeron comentarios a cada uno de los fragmentos divididos anteriormente, dando lugar a la codificación abierta.

**Figura 1:**
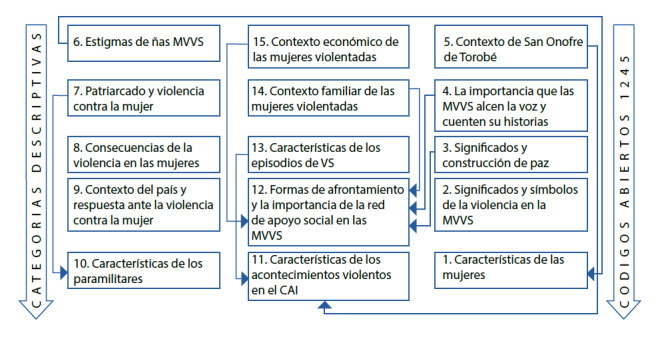
Categorías descriptivas.

Las entrevistas grabadas tenían una duración de entre 40 a 60 minutos de audio registrado cada una; en total se reportaron 1.245 códigos abiertos resultantes de los datos recolectados. Adicionalmente, estos códigos se compararon entre sí con el propósito de establecer conexiones logrando realizar una reagrupación por afinidad o similitud, lo que llevó a la construcción de 15 categorías descriptivas^12^ (Ver Figura 1).

Para la reagrupación se utilizó el programa Microsoft Office Excel, en la hoja de cálculo se organizó los códigos por cada interlocutora, y luego se adicionaron las reagrupaciones de los códigos de todas las interlocutoras entrevistadas. Luego de esto se rotularon los códigos con nomencladores, y se reagruparon, dándole un nombre a cada grupo de códigos, lo que llevó al establecimiento de las categorías descriptivas mencionadas (Ver Figura 1). Se establecieron interacciones entre las categorías descriptivas, con el fin de obtener una nueva guía de preguntas que validarán dichas relaciones y afirmaciones en la siguiente fase^12^.

### Categorías analíticas

**Figura 2:**
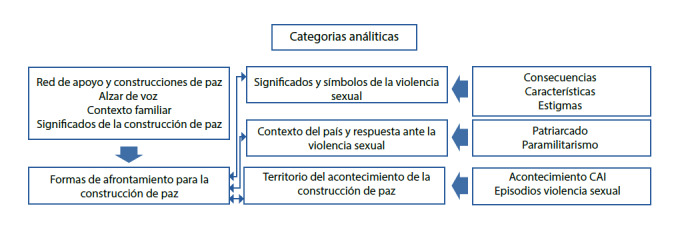
Segunda etapa: Codificación axial

En la segunda fase, teniendo en cuenta la nueva guía de preguntas (31) y la selección de un nuevo grupo de mujeres interlocutoras (muestreo teórico), se continuó con el proceso de recolección de información, dando inicio a la segunda fase y a la codificación axial del estudio. En esta se entrevistaron a cuatro mujeres interlocutoras, se lograron 716 códigos adicionales^12^. Para este proceso se analizó la extensión de los datos, las conexiones, las dimensiones, y las nuevas categorías analíticas (reduciendo las categorías descriptivas) (Ver Figura 2) que orientaron nuevos interrogantes, que a su vez guiaron el inicio de la construcción de una teoría sustantiva, descubierta y explicada de la mano de la metodología de la teoría fundamentada.

En la tercera fase, se ubicaron 3 mujeres con quienes se acordaron los encuentros. En estas nuevas indagaciones se observó la reducción de los datos en las categorías, ampliando conceptos teóricos y estableciendo nuevos vínculos entre las categorías. En esta ocasión se obtuvo la saturación teórica, ya que no aparecieron nuevas ideas ni conceptos que llevaran a generar un nuevo conocimiento. Por el contrario, se repetían elementos ya identificados, que permitieron reafirmar la construcción de la hipótesis, nutriendo las 15 categorías ya existentes con nuevos códigos, se logró la obtención de 432 códigos adicionales que llevaron a la configuración de un grupo de hipótesis^12^. (Figura 1)

**Figura 3:**
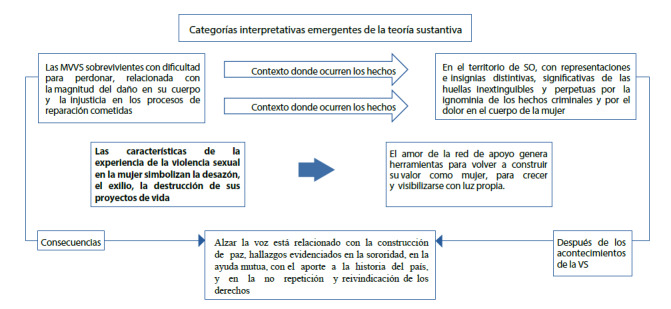
Construcción de la teoría sustantiva a partir de las categorías interpretativas.

## Resultados

Teniendo en cuenta las subjetividades de las mujeres, donde expresaron los significados de las violencias sexuales, reveladas en las categorías interpretativas (Ver Figura 3), se obtuvo el eje central final, expresado en “las características de las experiencias de violencia sexual en la mujer simbolizan la desazón, el exilio y la destrucción de sus proyectos de vida”, como resultado de las vivencias propiciadas por el conflicto armado; debido al malestar que manifestaron **las sobrevivientes por la magnitud de los daños en su cuerpo, y la injusticia en los procesos de reparación, ellas presentan dificultades para perdonar.** Las participantes pertenecen a un territorio golpeado por la violencia, en el que existen representaciones e insignias distintivas, significativas de las huellas inextinguibles, perpetuas por la ignominia de los hechos criminales y por el dolor en la mujer. Estas circunstancias experimentadas permitieron la identificación de estrategias de afrontamiento, tales como el amor de las redes de apoyo social que generan herramientas para volver a construir su valor como mujer, para crecer y visibilizarse con luz propia; de la misma manera, el hecho de alzar l a voz y ser escuchadas está relacionado con la construcción de paz, la sororidad, la ayuda mutua, el aporte a la historia del país, a la no repetición y a la reivindicación de sus derechos.

A continuación, se desarrollará la segunda categoría interpretativa resultante de la teoría sustantiva (Ver Figura 3): sobrevivientes con dificultad para perdonar, relacionado con el perdón, la magnitud del daño en su cuerpo y las injusticias en los procesos de reparación cometida.

Cuando se indagó por el perdón como un tema transversal a la violencia sexual en las interlocutoras, los testimonios revelaron que las mujeres víctimas de violencia sexual MVVS creen que es muy difícil perdonar, algunas afirmaron que es imposible olvidar la falta cometida contra ellas, pues fue tal la magnitud del daño causado por el victimario, que piensan que nunca eximirán de culpa por este hecho a quienes lo hicieron. Ellas manifestaron que pueden indultar la responsabilidad de otros actos, quizás de otros tipos de violencia, pero nunca perdonar los actos de violencia sexual.

Las sobrevivientes refirieron sentirse destruidas, dañadas en su integridad física y mental de forma permanente, siendo esta la razón principal de su incapacidad para perdonar. Estas mujeres eran conscientes de la importancia del perdón para su elaboración personal, y para su contribución a los procesos de paz y reconciliación; sin embargo, no quieren aceptar ningún vínculo o relación con el victimario, dada la injusticia perpetrada. Por otro lado, ellas suplicaron conocer la verdad de los actos cometidos y la justificación de la muerte de sus seres queridos; aducen que, para entrar en el camino del perdón y la reconciliación, los criminales deben revelar la verdad sobre los actos consumados, así como mostrar un arrepentimiento genuino por aquellos.

Las interlocutoras expresaron que el perdón es difícil dada la impunidad de los crímenes; también evidenciaron que la acción de perdón no tiene ningún sentido para ellas, pues es tanto el dolor y el sentimiento de burla experimentados, que su sufrimiento permanecerá por siempre. Adicionalmente, algunas mujeres narraron que no pueden perdonar, ya que sus victimarios actuaron de manera premeditada y con sevicia; esto se evidencia en los siguientes relatos:

*Sí señora, eso fue horrible lo que vivimos en ese pueblo, yo tuve que convivir con la persona que abusó de mí. Lo veía en la mañana, todos los días, tenía sólo 13 años. Él le dijo a mi mama que iba a prender la casa Esa zozobra. Hay secuelas. No, yo no perdono (E10LM).*

*Perdonar sí significa construir paz, yo no voy a perdonar. Porqué bañaron en sangre al pueblo. (E10SO).*

*Claro, uno nunca va olvidar, esas heridas y el recuerdo está ahí, lo que uno vivió en carne propia, eso está marcado (E11G).*

En el estudio realizado por Suárez Pinzón “Violencia de género y violencia sexual del conflicto armado colombiano^13^”, se hacen descubrimientos similares a los recolectados en esta investigación; pues el asesinato y la tortura son fenómenos cotidianos en las zonas de guerra en el país en las que existe predominio paramilitar. En estas zonas, las mujeres recuerdan las masacres presenciadas, los cadáveres, la prohibición de recuperar a sus muertos para darles sepultura, el terror y la huida de quienes perdieron todo por salvar su vida.

Lo que se evidencia en estos relatos es que los eventos todavía siguen allí, sin tramitación, lo que también ha demostrado la falta de garantías estatales para la reparación psicosocial de las víctimas. Algunas interlocutoras refirieron que no están preparadas para perdonar a su victimario, por el sentimiento de vergüenza y humillación, que se sienten limitadas para emprender sus proyectos de vida. Estas mujeres indicaron que ser víctimas de violencia sexual no tiene cura, ya que no hay tratamiento adecuado para el trauma y la pena moral causada por la agresión; esto se manifiesta en la siguiente narración:

*Todo queda en la cabeza, por lo menos a mí no se me ha olvidado, todos los días lo recuerdo, y ya va a tener 17años (E7KJ).*

Por otro lado, en reportajes de prensa se pudo corroborar lo referido anteriormente respecto a los hechos de violencia y dolor acontecidos en el pueblo de San Onofre. Este es el caso del reportaje de Laura Toscano titulado “El trapo, símbolo del dolor para un grupo de mujeres sucreñas^14^” en el que se muestra cómo las mujeres, con la mirada perdida y la respiración profunda colocaban el trapo sobre sus hombros, y se secaban las lágrimas en las exequias de sus difuntos. Es un elemento útil para secar las lágrimas de desesperanza por arrancarles lo más preciado de sus entrañas, un llanto de piedad; también sirve para cubrir los fragmentos esparcidos producto de la destrucción por una guerra, escondiendo en el trapo la profundidad del mar de lágrimas inconsolables en los ojos por la pena de su fallecido y por las violencias experimentadas.

Para las MVVS cada vez es más lejana la idea de perdonar, de olvidar, de sanar heridas abiertas; quizás por la poca disposición cognitiva de las víctimas para reducir los sentimientos negativos o sentir compasión hacia sus victimarios, por la rabia, se convierten en un obstáculo más para sanar, todo ello por la falta de procesos de reparación y de justicia que pudieran ayudar en esta tramitación. El camino hacia el perdón no representa infringir los derechos de las mujeres, no garantizarlos, y no cumplir con las leyes de la justicia, significa que no es solo enfrascarse y estar sediento en esa justicia restaurativa, este comportamiento dificulta el avance a cumplimiento de metas, de proyectos en la vida de las agredidas^15^.

El perdón es sinónimo de sanación, de terapia, transformación de comportamientos positivos, en no seguir pensando en la agresión y crear mayor destrucción, es una consigna de querer lo mejor para sí misma, en crecer para las personas que amamos, desear lo mejor a los otros, cambiando los pensamientos y sentimientos negativos y reducir de cierta forma el dolor, el sufrimiento, con la convicción de que vendrán mejores tiempos.^15^

El perdón no debe favorecer a la impunidad, borrando a la justicia, menos si el pacto es construir la paz, y para eso es inminente cumplir con los castigos, y no exonerar a los victimarios, más por las marcas tatuadas en los cuerpos de las mujeres, desde el despojo, la vergüenza, ultrajes, desarraigos, barbaries y la exclusión cometida en muchas de ellas^16^.

En los escenarios de conflictos armados, de violencia en contra de la mujer, se deben visibilizar estas acciones de injuria y de crímenes, para garantizar los derechos humanos, la no repetición del daño, y resarcimiento del dolor ocasionado. “No se le puede pedir a las víctimas de violencia que, en nombre de la paz y la reconciliación, perdonen a sus agresores y olviden, para que ahora “todos juntos”, en una sociedad desigual, cuya desigualdad se ha profundizado por el conflicto, que está en condiciones de exclusión o indigencia, o que no sabe siquiera donde están sus muertos, que sencillamente perdonen y se sumen al canto glorioso por la paz^17^”.

La acción del perdón no es quedarse quieto, sin decir ni hacer nada, y soportar aceptando el suceso. Perdonar, es más, es tener ese ímpetu de protesta, exigiendo lo que merecen, lo justo, siendo agentes de cambio de su realidad social, venciendo el miedo, hablando de su historia, dejando la rabia e impotencia atrás para visibilizarse como sujetos de derechos.^17^

En este sentido, las acciones del perdón se suman todas las mencionadas, aportando a la transformación social de las mujeres, reconstruyendo el tejido social, reparando a las sobrevivientes, dando paso a la reconciliación con ellas mismas, resarciendo el daño y el dolor^16^.

Por otro lado, “el perdón no puede ser compatible con el olvido: ¡Se perdona desde la memoria! Lo que sucede con el perdón es que el recuerdo deja de generar dolor y odio, se transforma en noticia de lo que no puede volverse a repetir. Pero también puede afirmarse que la impunidad dificulta procesos de perdón^17^”. Ahora bien, la gente reconoce que lo justo, la justicia y la reparación se concretan de una manera efectiva y clara en la no repetición de los hechos violentos vividos, es decir en la concreción de una paz estable, sólida y duradera^18^. El perdón es un proceso que deben vivir las personas, respetando sus ritmos subjetivos e, incluso, la decisión de no perdonar.

Una de las razones de las mujeres de no perdonar es por la falta o nula reparación, ellas manifestaron en sus relatos las injusticias durante los procesos, causados por demoras, trámites y gestiones consideradas innecesarias y que sólo las revictimizan. Mientras tanto, las sobrevivientes protestan con su silencio, anhelando un mañana mejor y una mejora en su calidad de vida. Estas dificultades pueden corroborarse en los siguientes testimonios:

*Yo considero que sí, como mamá soltera, no me ha tocado fácil, ha sido duro. Pues sí, es importante el apoyo del gobierno, aunque esa plata no va a cicatrizar la herida que me quedó, pero sí me ayuda en muchas necesidades (E7YP).*

*Sí, a mí me incluyeron en las víctimas de desplazamiento forzado por los papeles y me daban subsidio cada 4 meses, pero ya no me lo dan. Yo quiero por lo menos una vivienda digna, algo para darle de comer a mis hijos (E8YS).*

En estos relatos, la reparación económica aparece como una salida a los apuros económicos, pero también como una forma de legitimar sus testimonios y los daños causados por las fuerzas paramilitares cuando usaron sus cuerpos como botín de guerra. A las MVVS se les dificulta alcanzar el perdón ya que muchas de ellas no lograron alcanzar sus proyectos de vida debido a su condición de víctimas. Estas mujeres quieren progresar y brindarle una mejor calidad de vida a sus hijos, y para esto necesitan recibir la reparación y el resarcimiento, pues ello les devuelve su condición de legitimidad humana.

Asimismo, las interlocutoras exigen que se les garanticen sus derechos, pues varias sobrevivientes han padecido señalamientos y situaciones de discriminación por parte de personas del pueblo, ya que la mayoría de los habitantes de San Onofre conocen la historia de las mujeres. Ellas han tenido que soportar insultos y burlas que afectan su salud mental. Este tipo de escenario social da cuenta una vez más de las estructuras patriarcales, donde la mujer abusada es de alguna manera culpable de lo sucedido, donde persiste la paradoja de un cuerpo que le pertenece al más fuerte, pero una culpa que es solo suya. Los siguientes relatos ponen en evidencia lo dicho anteriormente:

*Las mujeres no tenemos calidad de vida, ustedes no han pasado por lo que vivimos: el calvario, el sufrimiento. Somos señaladas siempre, me tiran piedra, excrementos, carpetas, me hacen bullying, yo no soy culpable. (E10SO).*

Uno de los hallazgos demostró la precarización de las vidas de las jóvenes MVVS sanonofrinas, quienes siguen padeciendo constantemente diferentes formas de matoneo, señalamientos, rechazos y humillaciones que les impiden llevar una vida normal, lo que conlleva a repercusiones en su salud física, mental y ocupacional. Estos descubrimientos son similares a los presentados por la Consejería de Derechos Humanos de la Presidencia de la República, en los cuales se demostró que una de las estrategias de guerra usada por las bandas criminales es “causar deliberadamente que el grupo tenga condiciones de vida que están pensadas para llevar a su destrucción física por completo o en parte”^19^^,^^20^ esto debe interpretarse como un método de destrucción, donde “el perpetrador no mata inmediatamente a los miembros del grupo, pero que, a la larga, busca su destrucción física”^20^^,^^21^. Esto supone, por ejemplo, someter a un grupo de personas a una dieta de hambre, a la expulsión sistemática de sus casas y a la reducción de los servicios médicos esenciales al mínimo requerido^19^.

Las narraciones de las mujeres dieron cuenta de todas las injusticias experimentadas, ya que, sin tener la culpa de lo sucedido en sus vidas, continúan soportando malos tratos y atropellos por parte de sus vecinos por su condición de víctima, por el señalamiento como culpables por parte de quienes le rodean.

Adicionalmente, ellas manifiestan que el perdón en un camino largo y tortuoso, en el que se encuentran con muchos obstáculos y dificultades. Quizás para lograr perdonar necesitan ayuda profesional, o sólo tiempo, o tal vez haga falta otra vida para dejar de recordar el daño causado, para sanar sus heridas que no se borran. Igualmente, enfatizan en que la reparación del gobierno no es suficiente para resarcir el daño causado. Las narraciones de las mujeres expresan lo dicho anteriormente:

*Si señora, aunque esa reparación del gobierno que nos da por ser víctima no repara todo el daño que nos hicieron. pero bueno, peor es nada. (E11G).*

Los procesos de reparación de las mujeres víctimas de San Onofre no han sido satisfactorios, debido a la falta de celeridad en los mismos; esta situación se ha repetido en otros contextos, como se evidencia en el Informe Anual de la Comisión Interamericana de Derechos Humanos “el Gobierno está cercenando el derecho a la verdad, la justicia y la reparación, y distorsionando la realidad de un conflicto en el que todas las partes siguen cometiendo abusos contra los derechos humanos y violaciones del derecho internacional humanitario^21^”. De los principios formulados por la Comisión de Derechos Humanos para la protección y promoción de los mismos se concluye que toda víctima, tanto por la vía penal como por la civil, administrativa o disciplinaria, deberá contar con la posibilidad de acceder a una pronta y justa reparación; el artículo establece que “toda violación de un derecho humano da lugar a lo cual implica el deber del Estado de reparar y de dirigirse contra el autor^22^”

La reparación integral de las MVVS es necesaria para alcanzar la justicia y la construcción de paces; es aquella que reúne y potencializa los recursos disponibles para disminuir los riesgos vitales y las secuelas permanentes del conflicto armado. La reparación integrada, es el conjunto relacional de acciones que, enfocadas en resarcir el daño a las víctimas, busca la resignificación de los contenidos y efectos dolorosos de la guerra, o de otros hechos victimizantes, propiciando posibilidades de integración entre medios, recursos, procesos, instituciones, actores sociales y todos aquellos que puedan colaborar con la superación de los daños reales y potenciales^23^.

En otra publicación realizada por la Unidad de Víctimas llamada “En Libertad (Sucre), que resistió a la violencia paramilitar^24^”, se evidencia que el pueblo de San Onofre fue uno de los territorios escogidos en su programa Sujeto de Reparación Colectiva en Sucre, específicamente el corregimiento de Libertad; este programa es una muestra e iniciativa de sus pobladores como alegoría a la resistencia y a la paz. En el reportaje se evidencia el valor y el coraje de un pueblo para quitarse las cadenas con el fin de defender su territorio y conseguir la libertad, además de vencer el miedo a denunciar a los culpables, para que sus crímenes no queden en la impunidad.

En el reportaje del Ministerio del Interior, “Víctimas en San Onofre validan mejoras en atención en San Onofre^25^”, se indican los resultados de la evaluación de los procesos de atención y reparación integral a las víctimas, producto del proyecto de fortalecimiento institucional para establecer un plan de mejoramiento específico, iniciativa que fue desarrollada en el municipio de San Onofre.

Esta publicación es el resultado del desarrollo de políticas y programas en pro de las víctimas del conflicto armado, de la lucha de sus habitantes por la restauración de sus derechos y del afán para que se conozca la verdad de lo vivido en su pueblo azotado por la violencia. Se evidencia el camino hacia la luz en medio de la penumbra, de la claridad hacia la reconciliación, de que algún día no muy lejano encontrarán la armonía y la tranquilidad.

Adicionalmente, teniendo en cuenta el desarrollo de planes y programas establecidos para atender a las víctimas del conflicto armado en San Onofre y según un estudio que realizó la Universidad Javeriana, titulado “Plan Integral de Reparación Colectiva de Libertad (Sucre), expectativas y realidades de las víctimas^26^”, los pobladores del corregimiento de Libertad en San Onofre aceptaron el proyecto del Gobierno Nacional, convirtiéndose en el corregimiento piloto en el Programa de Reparación Colectiva. Para la realización de este programa, durante cinco años la Comisión Nacional de Reparación y Restitución (CNRR) elaboró el diagnóstico de los daños causados con ayuda de la población víctima del corregimiento Libertad, posteriormente, el estudio pasó a ser implementado por la Unidad de Atención y Reparación Integral a las Víctimas (UARIV) con la aparición de la Ley 1448 de 2011^27^^,^^28^^).^

Con el paso de 10 años, el Plan Integral de Reparación Colectiva Libertad (PIRCL) aún se encuentra en la fase de implementación y seguimiento, la cual contempla medidas con acciones específicas, efectuadas con el fin de garantizar el goce de derechos en salud, trabajo, educación, vivienda, atención psicosocial, entre otros factores. Los hallazgos reportados en el estudio anteriormente mencionado, realizado por la Universidad Javeriana, determinaron que el análisis del proceso de reparación colectiva fue prácticamente nulo en cuanto al logro de las medidas del Plan Integral de Reparación Colectiva (PIRCL) desarrolladas por la Comisión Nacional de Reparación y Reconciliación (CNRR)^26^^,^^29^; observándose que en 10 años no ha sido posible registrar un avance significativo en la consecución de lo establecido en el proyecto, evidenciando que los esfuerzos han sido insuficientes ya que no hay presencia del Estado en la comunidad.

En consecuencia, es importante resaltar que según las evidencias halladas en las narraciones de las voces de las mujeres de SO y el estudio de la Javeriana, no se ha llevado a cabo eficientemente el desarrollo de los proyectos de reparación, los cuales cuentan con innumerables deficiencias, causadas tal vez por falta de monitoreo y vigilancia del Estado, por la incapacidad de algunos profesionales en su operatividad, o por las dificultades propias del pueblo en materia de orden público y la presencia de grupos al margen de la ley.

En las narraciones de las mujeres se enfatizó en la compensación económica, indemnizaciones a las que tienen derecho por su condición de víctimas, y que sin embargo no son suficientes para reparar la magnitud del sufrimiento vivido. No obstante, el proceso de reparación es mucho más profundo, pues va más allá de la distribución de bienes y servicios materiales, que ellas quizás puedan pensar que solventarían sus necesidades. La reparación incluye áreas como el reconocimiento, el respecto a la dignidad de las mujeres víctimas, la garantía y protección de sus derechos que en muchas ocasiones siguen siendo violentados, incluso después del desplazamiento forzado causado por el conflicto armado. Las mujeres, además reclaman conocer la verdad de los hechos por parte de los actores armados, para que así los crímenes no queden en la impunidad y ayuden a las sobrevivientes a mitigar el dolor y a superar las secuelas que se encuentran en sus cuerpos de forma permanente.

Ellas piden saber dónde se encuentran los desaparecidos, los cadáveres de sus familiares asesinados, las circunstancias de los hechos, los argumentos de los victimarios, entre otros datos que aún son vedados. Por tanto, ellas tienen juicios valederos desde su percepción para no querer perdonar, regidas por el miedo inmutable que las persigue en todo momento y el repudio desmedido por parte de los demás, quienes las señalan y las condenan por ser víctimas de sanguinarios, perpetuando un esquema de vida patriarcal donde las mujeres siendo víctimas siguen siendo culpables.

Por otro lado, si la institucionalidad hubiese seguido los parámetros pactados en los documentos guías de actuación, tales como el Plan de Desarrollo del Departamento de Sucre de la época^28^, la historia sería diferente y muy probablemente el proceso de reparación hubiese tenido resultados positivos. En el documento mencionado, se subrayan afirmaciones como “asistir y reparar a las víctimas es construir paz”; lo que indica que con este Plan se pretendía asegurar la protección de los derechos humanos y mejorar las condiciones socioeconómicas de los habitantes del departamento. Es así como tras el paso de dos décadas desde los sucesos de esta guerra, aún no se cumplen las metas trazadas en la legislación colombiana para la reparación de las víctimas. Algunas de las ellas aún no cuentan con vivienda digna, seguridad alimentaria, trabajo estable o la culminación de sus estudios, quedando entonces la pregunta: ¿dónde están los entes territoriales que velan por los derechos de estas mujeres? Mientras tanto, ellas son heroínas en tener fe, por guardar la esperanza de alcanzar sus propósitos.

Para generar la reparación en las victimas debe haber perdón. “El perdón no anula la justicia, sino que es como la plenitud de la justicia. El perdón sin justicia se transforma en una virtud débil, floja, agotada y lastimosa, e incluso puede llegar a alcanzar la categoría de cruel, e injusta. El perdón con justicia podría ser uno de los pocos poderes morales que ayuden en la transformación del género humano^30^”. "Perdón para recordarlo todo sin dañar ni dañarnos". Establece un compromiso con la verdad y la memoria, porque en muchas ocasiones se oculta el crimen o se busca que se olvide para garantizar así su impunidad, criminalizando la memoria, como hemos visto recientemente en nuestro país con las resistencias a la memoria histórica, o infundir miedo y obligar a olvidar, ocultar los hechos y destruir las pruebas^30^.

## Conclusiones

Esta investigación permite reconocer el valor de la memoria colectiva para la gestión del conocimiento en salud pública, para el reconocimiento de las víctimas y las respuestas del Estado para recuperar la salud mental de las mujeres afectadas.

La recuperación de la memoria de las víctimas se halla en íntima relación con la protección y garantía de los derechos de las mujeres a través de la justicia, participación y movilización social, interés también de la salud pública, en tanto se develan las inequidades en salud y las causas determinantes de esta situación para un accionar ético y político.

Las mujeres víctimas de la violencia sexual fueron culpabilizadas de esta por la sociedad, lo que se expresaba en los señalamientos, en el repudio, y en la estigmatización, esta falta de solidaridad, o en la indiferencia de sus vecinos con malos tratos.

Los testimonios de las MVVS revelan los significados de la violencia sexual, el hecho de que es muy difícil perdonar, por la imposibilidad de olvidar el acto de horror cometido contra ellas, por la magnitud del daño causado, por pensar que nunca eximirán de culpa a los criminales que destruyeron su vida por el hecho cometido. Aducen que, para entrar en el camino del perdón y la reconciliación, los vándalos deben revelar la verdad sobre los actos cometidos, así como mostrar un arrepentimiento genuino.

Estas mujeres indicaron que ser víctimas de violencia sexual no tiene cura, ya que no hay tratamiento adecuado para el trauma y la pena moral causada por la agresión. Hay falta de compasión por la impotencia y los sentimientos de culpa que obstaculizan la sanación, los procesos de reparación y de justicia para la tramitación y resarcimiento del dolor.

### Límites

Durante el trabajo de campo no había libertad y seguridad tanto para las interlocutoras e investigadora, para la expresión verbal de la vivencia experimentada. Hubo paros armados, olas de asesinatos que impidieron a la investigadora entrar a San Onofre Sucre, lo que hizo que las participantes tuvieran que trasladarse hacia lugares más seguros. La pandemia en la segunda y tercera fase de recolección de información obstaculizó la recolección de información de manera presencial. Ninguno de estos eventos afectó la calidad de los resultados.
